# Stronger together: midwifery twinning between Tanzania and Canada

**DOI:** 10.1186/s12992-018-0442-x

**Published:** 2018-12-13

**Authors:** Rachel Sandwell, Deborah Bonser, Emmanuelle Hebert, Katrina Kilroy, Sebalda Leshabari, Feddy Mwanga, Agnes Mtawa, Anne Wilson, Amelie Moritz

**Affiliations:** 1 Canadian Association of Midwives, 2330 rue Notre Dame, Suite 300, Montreal, QC H3J 1N4 Canada; 2 Midwives Collective of Toronto, 1203 Bloor St West, Toronto, ON M6N 1H4 Canada; 3 School of Nursing, Muhimbili University of Health and Allied Sciences, P. O. Box 65169, Dar es Salaam, Tanzania; 4 Tanzania Midwives Association, P.O. Box 65524, Dar Es Salaam, Tanzania; 5 Tanzania Nursing and Midwifery Council, Kaluta Street, P. O. Box 6632, Dar es Salaam, Tanzania; 6 Fondation Sanofi Espoir, 262, Boulevard Saint Germain, 75007 PARIS, France

**Keywords:** Midwifery, Capacity-building, Professional associations, Twinning, Partnerships

## Abstract

This article describes a twinning relationship between the Canadian Association of Midwives (CAM) and the Tanzania Midwives Association (TAMA). It argues that the twinning relationship strengthened both associations. The article briefly reviews the existing literature on professional associations and association strengthening to demonstrate that professional associations are a vital tool for improving the performance of healthcare workers and increasing their capacity to contribute to national and international policy-making. It then suggests that midwifery associations are particularly significant given the frequent professional marginalization of midwives. The article then describes in depth the relationship between CAM and TAMA, highlighting the accomplishments of the twinned partners, and analyzing the factors that contributed to the success of the relationship. The findings demonstrate that twinning can successfully strengthen associations, increasing their ability to support their membership, care for the public, and shape national policy-making. The article therefore proposes twinning as a successful and cost-effective model for encouraging the growth of the midwifery profession.

## Background

This article describes and evaluates one twinning relationship between two midwifery associations, the Tanzania Midwives Association (TAMA) and the Canadian Association of Midwives (CAM), to argue that association twinning can be a powerful tool to strengthen the midwifery profession in different national contexts, with varying healthcare challenges and professional needs.

**Table 1:** Highlights from Umoja Strategic Plan 2011, and Status as of 2018 [39]

Strategic Objective	Activities	Status 2018
#1 Strengthen TAMA & Administrative Capacity	Raise funds for Umoja	Complete – project funding
	Increase staff for each association	TAMA: From 0 to 5 CAM: From 2 to 19
	Increase membership of each association	Both increased substantially.
#2 Strengthen TAMA & CAM’s ability to influence maternal newborn child health policy	Strengthen each association through revised constitution and membership policy, and increase visibility of Umoja relationship	Complete
	Increase visibility of midwifery to policy makers in Canada and Tanzania	Complete
	Increase midwifery representation in different national and international fora	Complete
#3: Strengthen midwives capacity to provide quality care in Tanzania and Canada	Needs assessment on emergency skills	Complete
	Emergency skills trainings run by TAMA	Complete
	Emergency skills for rural and remote midwives in Tanzania and Canada	Complete
#4: Facilitate collaboration on midwifery research, education and practice	Develop twin pairs between Tanzania and Canada	Complete (partial)
	Identify areas of collaborative research	Complete (partial)

The article first introduces the current global maternal and reproductive health context, with particular attention to the contributions midwives can make to improving health outcomes. It then discusses the roles professional associations can play in supporting access to quality reproductive healthcare. It then describes association strengthening practices, assessing the reported efficacy of association twinning as a strategy for strengthening healthcare professionals’ capacities. It then analyzes the specific relationship between TAMA and CAM to explore how and why twinning worked to strengthen both organizations.

The global south, and the African continent in particular, is widely recognized to be facing a crisis of human resources for health – in the face of a rapidly growing population, there is an insufficient number of healthcare workers available, and many of the healthcare workers that are present have an inadequate skill level [1–3]. While Africa bears 24% of the world’s disease burden, it employs only 3% of the world’s health workforce [4]. In Tanzania, it is estimated that only 64% of reproductive healthcare needs are met, and the continuing high rates of maternal and neonatal mortality testifies to the inadequacy of existing healthcare systems, and the obstacles healthcare professionals face in providing care [3, 5].

International organizations including the UNFPA (United Nations Population Fund) and the International Confederation of Midwives (ICM) have emphasized that midwives are a vital resource for the struggle to improve access to reproductive and sexual healthcare. Midwives trained and regulated to international standards can provide 87% of the necessary care for mothers and babies, including antenatal care, birth, and postnatal care for mothers and infants; they can also provide a broad spectrum of sexual and reproductive health and rights services to diverse populations, including contraception and post-abortion care [2]. Midwives can play a vital role in meeting Sustainable Development Goal (SDG) 3.1, reducing maternal mortality ratio to less than 70 per 100,000 live births, and Target 3.2, reducing neonatal mortality to 12 per 1000 live births [6]. However, research indicates that there continues to be a significant shortage in midwives worldwide, and that practicing midwives frequently report feeling under-prepared and under-supported in their work [7].

Although Canada is a wealthy country with a strong healthcare system, midwifery is a young profession in the country. Canada only legalized and regulated midwifery in 1994, after significant activism from the women’s health movement and from Indigenous midwives, and there are still fewer than 2000 practicing midwives in the country, although that number is increasing annually. Midwifery is not accessible everywhere in the country. Midwives have therefore struggled for recognition as healthcare professionals capable of providing care and as experts capable of contributing to national and international healthcare policy [8–10].

Twinning has been proposed as one way to strengthen the skills and capacities of midwives, but most studies of twinning have focussed on relationships between individuals within officially twinned organizations [11]. This article argues first that professional associations are vital for improving the quality of midwifery care in a sustainable way in any national context; and secondly that long-term twinning relationships can be a powerful tool to build capacity in professional associations in the global north and the global south. In highlighting the evolving relationship between TAMA and CAM, it emphasizes that the twinning relationship was not merely beneficial for TAMA, but for CAM as well. This case study challenges conventional models of development to argue that global south-global north partnerships can be enriching for all parties involved.

### Why professional associations matter

Associations are a vital component of the midwifery profession. The ICM emphasizes three pillars of midwifery – education, regulation, and association [12]. As former UNFPA Director Babatunde Osotimehin remarked, “If one of these pillars is weak, the whole of midwifery will be weak” [13]. Associations have a keystone function for the profession: it is associations that act as a focal point for midwives, ensuring the quality of the training and support available to pre-service and in-service midwives, and acting as a voice to represent the profession at the national level. Strong associations can safeguard the quality of education and regulation in a given national context. Despite their importance, midwifery associations have received little attention either in the academic literature or from funders, aside from the ICM. The ICM has played a major role in raising the profile of midwifery in global health, ensuring the voices of midwives are heard at the policy level, and in reinforcing the capacities and skills of midwives around the world. While international efforts have been relatively quick to fund educational programs for midwives around the world, less attention has been devoted to regulation and association.

Because midwifery is already a marginalized profession, with midwives often viewed as either merely a specialized nurse or a doctor’s assistant, midwives face particular challenges in contributing to national policy and independently regulating their profession [14]. Although midwives and nurses provide an estimated 80% of all healthcare services worldwide, these professionals, and especially midwives, have a more difficult time in having their voice heard at a national policy level [15]. This lack of influence persists despite compelling evidence of the benefits that high-quality and well-regulated midwifery care can provide [16]. In particular, the lack of a professional association to unite midwives can lead to the implementation of poor quality programming, for example the replacement of skilled healthcare workers with poorly-trained general purpose workers [17, 18]. Without a professional association to advocate for regulation, midwives may be unable to practice to their full scope, meaning midwives could be forbidden from performing lifesaving obstetrical or neonatal interventions. Equally, midwives may not be able to collect or share data, participate in maternal death reviews, or have their own practice monitored or evaluated by other midwives [18]. Research has found that national governments in some cases do not consult with midwifery professional associations when setting maternal and child health policies, despite the expertise the associations could contribute. [19]. Midwifery associations can both provide support to practicing midwives, ensuring they receive adequate training, and can represent the interests, and convey the expertise of, their membership at the national and international leadership and policy level [20–22].

Because midwifery is in general a neglected profession, many aspects of midwifery remain under-researched [17, 19]. This may account for the lack of research on the role and potential of midwifery professional associations, as well as the shortage of projects aimed at building association capacity. However, there is an existing body of work on other health professional associations, and the findings are directly relevant to the midwifery profession. Associations of obstetricians and gynecologists in particular have militated for a more active and engaged role for their professional associations. Members of these associations have outlined a vision for a role for associations beyond merely sites for sharing knowledge inside the profession. Instead, they have argued, professional associations can contribute directly to improvements in health care, particularly through offering professional expertise to guide governmental decision-making about maternal and reproductive healthcare [23]. Associations can focalize the expertise of their members, practicing healthcare professionals, and relay that expert knowledge to national and international policy-makers and to the general public [24]. Health professional associations can therefore serve a vital public health function in two directions: they can “educate up”, helping to inform and guide policy makers; and they can “educate down”, helping to share information with the general public [23–28].

Inspired by this more activist vision of their role, professional associations, most notably the Society of Obstetricians and Gynecologists of Canada (SOGC), and the International Federation of Obstetricians and Gynecologists (FIGO), have developed programs to assist their fellow professional associations in the global south achieve similar strengths and similar results. The SOGC’s Organizational Capacity Improvement Framework (OCIF) was the first tool developed by a professional association to help bolster the organizational capacity of its fellow associations, and has been used in countries in Africa, Latin America, and Asia. More recently, and building on the SOGC model, FIGO launched its LOGIC (Leadership in Obstetrics and Gynecology) initiative. Over a period of several years, FIGO worked with member associations in eight countries in sub-Saharan Africa and Asia; results included association-led campaigns to roll out lifesaving medications, the development of strategic plans, advocacy plans, and increased participation in national policy-making [29–31]. According to FIGO’s assessments of their projects, the FIGO LOGIC association strengthening activities led to stronger health systems in these contexts [32, 33].

The ICM, UNFPA, and other international organizations have therefore focussed their energy in recent years on building the capacity of midwifery associations, arguing that “capacity building is critical to scaling up the midwifery workforce and improving maternal and child health” [20]. Capacity is broadly understood as being the catalyzation or actualization of resources a group or country already has – capacity development rests on the idea that a country does not need external resources, but merely assistance in transforming their existing people, organizations, and societies [15]. Twinning has been proposed as one way to effect such catalyzation.

However, as Dawson et al. remark, there has been very little evaluation of how capacity building can be done with midwifery associations [20]. Indeed, little research exists on the outcomes and efficacy of transnational partnerships in the health professions; scholars and health professionals have called on organizations who have participated in such partnerships to analyze and share their results, to better inform partnership practice [4]. This article therefore examines one successful twinning relationship to analyze how and why it worked, and what benefits it provided.

## Main text

### Why twinning

Twinning has been defined as a “cross-cultural, reciprocal process where two groups of people work together to achieve joint goals” [11]. In the field of global health, institutions in the global south and global north have used twinning to build skills-sharing relationships. Increasingly, development funders encourage such relationships, particularly between global south and global north organizations. Many of these relationships have included an institutional partnership, for example between universities or other health training institutions, or between professional associations, but have in practice focussed on person to person twinning. In such cases, the larger institutional partnership organizes a series of “twins”, with one twin in the global north and one in the global south. These twins have a series of individual meetings, usually by telephone, skype, or other electronic communication technologies, and share skills, experiences, and information. They may also undertake projects together, such as devising a joint training program or fundraising activity [4, 34–36]. Such initiatives have been lauded as cost-effective [36]; they also emphasize mutual learning, insisting that the global south partners can bring useful knowledge and information to the exchange, rather than just being passive participants [34, 35].

The relationship between CAM and TAMA is unique in the context of other twinning relationships, for two reasons: first, although the relationship began with project support from the ICM, and has involved important collaborations on projects, the twinning is consciously understood by all parties to transcend any one project. Therefore, it represents a long-term capacity building exercise. Secondly, distinct from most other twinning projects, the focus has been on each association, rather than person to person twinning: while in other twinning projects, much emphasis was placed on the relationships generated between individuals in each association, in CAM and TAMA’s case, the focus was on the relationship between the two associations, mediated by a formal steering committee. This steering committee, named Umoja (Swahili for ‘unity’), consisted of members from each association, chosen as representatives. It managed all activities of the twinning. The activities undertaken in the relationship, therefore, focussed on strengthening both associations, not individual members. Studying this twinning relationship provides an important case study highlighting how two professional associations can collaborate for their mutual benefit in a sustainable way. Although an external evaluation of the CAM-TAMA twinning process has not been conducted, objective analysis of the accomplishments of the twinning relationship, measured against its intended goals when it started, demonstrate that the partnership has been highly effective in accomplishing its goals.

### Umoja: TAMA and CAM’s relationship

#### The relationship

TAMA and CAM were formally “twinned” by the ICM in 2011. In this context, twinning meant that the two associations signed a formal partnership agreement, formed a joint steering committee, the Umoja committee, and created a joint strategic plan, under ICM supervision. In the eight years since this relationship began, both CAM and TAMA have grown substantially in size, visibility, influence, and administrative capacity. Both organizations increased their staffing, budgets, and numbers of projects. They have also increased their capacity to provide leadership on maternal and reproductive health policy and practice in their respective national contexts.

#### Origins

The ICM started TAMA and CAM’s relationship, providing key support by introducing the two associations, and providing funding for representatives from both organizations to meet in person on three occasions. The ICM facilitated preliminary introductions at the ICM Durban (South Africa) conference in 2011, then hosted formal launch meetings in the Hague (Netherlands) in 2011, and brought Umoja members together at the Regional Conference of the ICM in Hanoi (Vietnam) in 2012. The ICM facilitated this twinning in the context of a larger initiative aimed specifically at association strengthening, which also saw three other twin relationships created (Mali-Switzerland, Papua New Guinea-Australia, and Vietnam-Japan). [37] At the initial 2011 meeting in the Hague TAMA and CAM developed their first strategic plan, with guidance and support from ICM staff. Recalling this first meeting, TAMA and CAM members emphasized that the funding from ICM for in-person meetings, and the practical support from ICM staff, were significant factors for the successful start of the twinning relationship [38].

#### Strategic plan: Goals and accomplishments

Umoja participants recalled that despite the many apparent differences between the Tanzanian and Canadian contexts for midwifery, the two associations shared a remarkable number of features and goals. In 2011, both associations were relatively new: TAMA had been formed in 1996, while CAM was formed in 2001. Both organizations were largely voluntary; in 2011 CAM had only two full-time staff, while TAMA had none. Both wanted to revise their constitutions, to expand their own administrative and staffing capacity, to seek external funding, and to raise their visibility and profile with their respective national governments. Equally, both had relatively small numbers of members spread across a large geographic territory, and both had an interest in supporting midwives working in rural and remote areas [38].

The Table below sets out initial objectives of the Umoja Strategic Plan, developed in 2011, in the first and second columns. The third column represents the Umoja Steering Committee’s assessment of their achievements as of 2017–2018, when a new strategic planning process began. As the Table demonstrates, the majority of the strategic objectives from 2012 were completed by 2018 (Table 1).

#### Project funding: Improved service delivery for safer motherhood, 2013–2018, Fondation Sanofi Espoir

As part of the Strategic Plan, Umoja members had committed to seeking funding for joint TAMA and CAM initiatives. In 2013, Umoja members, with support from CAM staff, submitted an application for funding via Fondation Sanofi Espoir’s “Midwives for Life” initiative. As Umoja member Dr. Sebalda Leshabari recalled, “we worked day and night to get that application in.” The application was successful: the Fondation Sanofi Espoir selected TAMA-CAM’s project, titled “Improved Service Delivery for Safer Motherhood”, for funding. All members of the Umoja Steering Committee agreed that TAMA and CAM’s success in obtaining this funding was a key part of cementing and expanding the Umoja relationship. [38].

The project’s main focus was the development and delivery of an Emergency Skills Training program, for midwives by midwives. The training was jointly developed by Tanzanian and Canadian midwives, and jointly delivered by Tanzanian and Canadian volunteers in six rural areas in Tanzania. The five-day training, known as “Midwives Emergency Skills Training” (MEST), could replace the much longer emergency obstetrics training already offered in Tanzania, which at six weeks in length was difficult for practicing midwives to attend. MEST was based on the Canadian Emergency Skills Workshop developed by the Association of Ontario Midwives, and specifically focused on skills for coping with emergencies, recognizing that many practicing nurse-midwives in Tanzania had only received training in uncomplicated birth. Canadian midwifery is an autonomous profession, and many Canadian midwives work outside of hospital or clinic settings, including in rural and remote settings; their training therefore emphasizes clinical decision making rather than technologically-based interventions, making it uniquely applicable in developing world contexts [40]. This skill-set meant Canadian midwives could effectively contribute to developing a training to be used in Tanzania, despite the differences in the context. Equally, the success of the training depended on the full participation of Tanzanian experts, most of whom were midwifery educators in the country’s university system. These midwifery experts were familiar with dealing with some emergencies that Canadian midwives rarely encounter and were also well-versed in local needs and cultural contexts, as well as in the existing Tanzanian curriculum. Their guidance therefore ensured that the training was appropriate and relevant to the context.

Once the program was developed, the training sessions were launched; the project ultimately trained 520 midwives between 2013 and 2018, in six rural regions of Tanzania. The project also worked to re-engage retired midwives, facilitated collaboration with community health workers, and supported advocacy with the government for increased rights and recognition for TAMA. Phase II of the project also engaged consultants from ICM to conduct “train the trainer” workshops with Tanzanian midwifery educators on competency-based education, to ensure that Tanzanian educators are able to educate and supervise students to an international standard. Educating educators will stabilize the gains made by the project.

The funding from Fondation Sanofi Espoir enabled Canadian midwives and CAM staff to travel to Tanzania to launch the project. It also funded Canadian midwives to travel to Tanzania to meet with their Tanzanian counterparts to develop the MEST training collaboratively, ensuring the project used local expertise and responded to local needs in a culturally appropriate way. The project enabled TAMA to hire their first staff, a Project Officer and Assistant, as well as a Finance Officer; CAM was also able to hire a part-time Project Officer and Finance Officer. The project helped CAM and TAMA share resources and expertise. For example, the project funded a three-week financial management and reporting workshop for TAMA staff and executives, led by CAM’s Director of Finance. Because CAM and TAMA midwives paired together both to develop and to execute the MEST trainings, the two associations had the opportunity to share and transfer midwifery expertise between their members.

TAMA gained significant organizational experience through managing the logistics for all the trainings. This process involved working closely with rural regional and district health administrators to arrange the trainings, and get permission for practicing midwives, who are employees of regional and district health authorities, to attend the sessions. TAMA managed all Tanzanian expenses and accounting.

CAM equally gained new management experience through their part in the project. CAM facilitated the attendance of Canadian volunteer midwives, and managed financial relationships with the donor. Through these processes, CAM staff and executives acquired new capacities in working across multiple foreign currencies, hiring staff with new skillsets, and training midwife volunteers to do overseas work in a safe and appropriate fashion.

By all measures, the ISDSM project was a success. Its Phase 1 was evaluated in 2015, after the project had trained 300 midwives; external consultants conducted document review, individual and group interviews, and site visits. The consultants found that the project had delivered high-quality training in a cost-effective manner, and their report recommended extending the project into a Phase II [41]. Fondation Sanofi Espoir concurred, and funded a Phase II, which began in December 2016 and concluded in September 2018.

Umoja members concur that the ISDSM project was crucial both for solidifying the relationship between TAMA and CAM, and for strengthening the individual capacities of each organization.

### Shared Project Management: Impacts on the Umoja relationship and each association

#### Strengthening the partnership

Running a project together both enabled and necessitated more communication and a closer working relationship between TAMA and CAM. Staff and executive members from CAM traveled to Tanzania for project start up meetings, and to create the MEST trainings. TAMA members were also able to travel to Canada to participate in meetings and conferences, in particular the ICM Congress held in Toronto in 2017 (hosted by CAM). Staff and executives from both associations met regularly by Skype and telephone in order to discuss the partnership and project implementation and management. The relationship became a financial one, requiring both organizations to build their capacity to manage financial communications and reporting. The pre-existing relationship, beyond the project partnership, helped ease the process of developing these tools. The capacities that TAMA and CAM developed together helped pave the way for future shared projects: the two associations now collaborate on two additional internationally-funded projects, and in 2018 submitted another joint project application.

#### Strengthening each association

Both TAMA and CAM experienced significant transformation since the beginning of the ISDSM project, and both emphasize project’s role in catalyzing these changes.

#### Staffing

Both organizations have more than tripled their staff sizes. For TAMA, the ISDSM project allowed them to hire their first staff. Prior to the project, the TAMA office had only been open weekends or after working hours, as the TAMA Executive were there only when they could be, while balancing their full-time work. With full-time project staff, the office can be open throughout normal working hours, and the office has become a site where many visitors gather to hear about TAMA’s work. It has begun to serve as a focal point for interested stakeholders, international visitors, and advanced midwifery students. TAMA now has six staff, while CAM has nineteen.

#### Visibility

The ISDSM project raised the profile of both associations, causing international NGOs and government agencies to take notice of TAMA and CAM’s work.

TAMA gained significant visibility as their staff, executive, and volunteers travelled to the six project regions to arrange trainings. Regional and district health administrators had to grant permissions for the trainings, and release midwives from their duties to allow them to participate. Through this process, these administrators became aware of TAMA’s work. Umoja members recalled that at the outset, local administrators had little knowledge of TAMA and were skeptical about releasing midwives to attend the MEST trainings. But once they attended the trainings themselves, and saw the results in their own midwives after the trainings, administrators took a much greater interest in TAMA, and also gained understanding of midwifery as a profession distinct from nursing [38]. This regional and district awareness will make it easier for TAMA to develop further projects in collaboration with local administrators; it may also make it easier for TAMA in the future to advocate for more recognition for the profession of midwifery and for the work of rural midwives.

TAMA also gained visibility at the national level. They successfully had the MEST training reviewed and approved by the relevant ministerial authorities. They have since been invited to participate in national technical working groups on maternal and reproductive health topics, including Maternal Death Reviews and consultations on national policy guidelines. In these ways, TAMA Executives are able to use their expertise to influence national policy making. TAMA has also gained recognition from international and local NGOs. They now collaborate with the UNFPA in Tanzania, including running trainings with them. In collaboration with CAM, they are working with the international NGOs CUSO and Jhpiego. They also recently received funding to lead their first project, from Laerdal Global Health, in collaboration with the International Confederation of Midwives (“50,000 Happy Birthdays.”). TAMA are also beginning the process to have their MEST training certified by the Tanzanian Nurses and Midwives Council (TNMC); this will mean midwives doing the training receive formal “Continuing Professional Development” credit, now necessary for renewing their professional license. If MEST gains this recognition, it will give both the training and TAMA significant sustainability, as the trainings will continue even without external project funding.

CAM also gained governmental recognition as a result of the project. Canadian Umoja members recalled that once CAM had their first international project, and in particular once they could demonstrate their successful relationship with TAMA, Canada’s foreign affairs and development ministry (now known as Global Affairs Canada) took CAM more seriously and CAM could more easily get meetings with government representatives. CAM was now treated as a viable partner in the development of international initiatives and the value of midwifery in the global context was more widely understood by funders. This connection aided CAM in developing further international projects, of which CAM now has five. But Umoja’s Canadian members are also confident that CAM’s international work, through Umoja and the ISDSM project, helped raise the profile of midwifery nationally. Midwifery is still an under-appreciated profession in Canada, and CAM is still advocating to build the profession inside Canada, working to ensure there are midwives available throughout the country. CAM members observed that governmental representatives, impressed by CAM’s global work with TAMA, seemed more receptive to midwives’ national role within Canada.

### Challenges

The main challenges Umoja faced, similar to the experience of most international twinning relationships, was communication. Neither skype, nor telephones, nor other internet-based communication technologies function at all times, and for the Tanzanian members, a lack of access to necessary technology and an unreliable electrical power grid sometimes made meetings hard. Planning meetings across large time differences was also a challenge (central Canada and Tanzania are seven hours apart). In addition, it was difficult to accommodate Umoja members’ busy schedules. All were working full-time, many with multiple responsibilities. Many of the Canadian participants were full-time practicing midwives, meaning they were frequently called away to attend births, which could impede planned meetings. All Umoja members agreed that maintaining the partnership was more difficult when in-person meetings were not possible.

Also, contrary to other twinning programs, in the case of Umoja, it was the person to person twinning initiative that failed. While other projects have reported relative success with individual twinning initiatives [34–36], in the CAM TAMA case, this was the weakest aspect of the collaboration. The project did attempt to pair a number of practicing Tanzanian and Canadian midwives, but these relationships faltered and never took root. Members attributed this lack of success to the lack of financial support for the program – there was no funding to support a paid coordinator, and practicing midwives in both countries were too busy to self-coordinate.

## Discussion: What made Umoja succeed?

What accounts for the durability and longevity of the twinning relationship between these two associations? Given the benefits the relationship has yielded for both partners, it is useful to understand what particular factors contributed to its success.

### Organizational similarities

Both TAMA and CAM were small and relatively young in organizational terms in 2011. This meant both were ambitious, and eager to grow. At the same time, they were both sufficiently established that they had organized governance structures, which enabled growth. Both had existing constitutions, procedures and policies, and well-established boards of directors. Both had clear vision and mission statements, and both were familiar with international midwifery through their connections to the ICM. It is possible that these similarities helped to forge a strong sense of connection. In organizing the twinning, ICM facilitators emphasized the mutuality and reciprocity of the relationship – the phrase which Umoja members recalled ICM facilitators repeating was, “there was no big sister and little sister – the two organizations were twins.” This reciprocity may have been easier to attain given the real organizational lifecycle similarities between CAM and TAMA. Equally, the reciprocity meant that both organizations were concretely benefiting and growing from the relationship, which kept both involved.

### International climate

The international global health climate and trends in donor priorities had a favourable effect on the initiative. In 2010, Canada’s government hosted a G8 meeting; there, “the Muskoka Initiative” was launched, a package of funding for international maternal, newborn, and child health, which called on G8 countries to commit to a total of $5 billion of additional funding in these areas [42]. To this, the governments of the Netherlands, New Zealand, Norway, the Republic of Korea, and Switzerland, as well as the United Nations and the Bill & Melinda Gates Foundation, which brought the total commitment to $7.3 billion USD. This initiative came in response to the growing international realization that, despite success in other areas of global development, maternal and neonatal health were still suffering and the Millennium Development Goals in these areas would not be reached. Internationally as well, midwifery was gaining greater recognition, thanks in part to advocacy work by ICM. This context meant that funders and international stakeholders such as the Fondation Sanofi Espoir were receptive to midwifery projects; this positive climate likely helped ICM to obtain funding for the twinning initiative.

### Personalities

Umoja participants also reported a more unquantifiable connection – they described that from the first project meeting in The Hague, they easily established an interpersonal rapport. They remembered that they seemed to be laughing much more than the other participants. They recalled that from the outset, they seemed able to have a lighthearted approach to the inevitable cultural miscommunications that occurred, and an easy social bond [38]. Some of this is simply the result of the personalities involved. But structural factors may also have played a role in shaping this relationship.

#### International outlook: Both the Canadian and Tanzanian participants had experience with international and cross-cultural work

The Tanzanian participants were all high-profile professionals; the majority of them had some overseas work experience, including completing degrees in universities outside Tanzania. They held positions of authority in Tanzania, working in leadership capacities in the education sector, in government, and in international NGOs. In this course of these experiences, they had gained strong awareness of international medical circuits of knowledge, work, and opportunity. This familiarity with working across cultural difference and in multiple national and international contexts may have facilitated the rapport they were able to build with the Canadian partners. The Canadian participants were less experienced in cross-cultural overseas work and education. However, all of them were highly active in their provincial and national midwifery associations, which brought them into contact with the ICM and the concerns of global midwifery, for example through attending the triennial ICM conferences which assemble midwives from around the world. This experience may have facilitated the Canadians’ experiences with cross-cultural communication. In addition, Canada contains multiple languages, including most notably English and French. The Canadian Umoja members were a mix of native English and native French speakers – as the Canadian national association works as bilingually as possible, members were familiar with trying to work in their second language, whether English or French, which may have increased their skills at cross-cultural and cross-language work.

#### The Canadian model of midwifery care

Another possible contributing factor may lie in the Canadian approach to midwifery, and the profession’s model of care. Midwives in Canada are taught to emphasize partnership throughout their work with pregnant and birthing clients, rather than to regard themselves as medical experts in a hierarchical relationship with their patients [40]. This mindset may have assisted the Canadian participants in approaching their Tanzanian colleagues with an open mind, receptive to the mutual learning and reciprocity intended by the twinning model. Canadian midwives also work flexible schedules; most are on-call for extended periods, rather than working fixed schedules. They learn to respond to the exigencies of situations. This context may have helped the Canadians respond effectively when meeting times and plans had to change due to logistical challenges.

### Project and relationship funding

Finally, funding, both for the initial twinning relationship and for the shared project, played an essential role in supporting the partnership. Given the limitations of communications technology, and the important role face to face meetings played in building rapport, it seems funding is essential. All participants agreed that the strength of the partnership waned at times when funding was not available to facilitate in-person meetings. This factor suggests that electronic-based meetings were not sufficient, and that some sustained funding is necessary to support twinning relationships. The relatively modest funding required can be considered cost-effective in comparison with other capacity-building exercises.

## Conclusion

Both TAMA and CAM have grown substantially through their twinning relationship. The twinning experience of Umoja demonstrates that twinning can be a successful way to strengthen midwifery professional associations. The participants report that the experience was dynamic and rewarding, and that both professional associations grew and increased their capacities in ways that could not be foreseen at the outset of the project. In turn, strengthened professional associations can better support practicing midwives, can contribute to national maternal and child health policy, and can help to guide educational requirements for pre-service and in-service training for midwives. Strong and active midwifery associations can improve the care available to pregnant people, parents, and young children, thus improving reproductive health and rights. The mutual and collaborative nature of CAM and TAMA’s twinning relationship challenges conventional models of international development, which tend to assume that aid flows unidirectionally from north to south. In the case of TAMA and CAM’s relationship, both sets of participants have grown together. Twinning thus provides a powerful model both for improving health systems and for contesting the hierarchies that too often shape interactions between global south and global north civil society organizations.

## Abbreviations

CAM: Canadian Association of Midwives
FIGO: International Federation of Gynecology and Obstetrics
ICM: International Confederation of Midwives
SOGC: Society of Obstetricians and Gynecologists of Canada
TAMA: Tanzania Midwives Association
UNFPA: United Nations Population Fund
